# Supplementary material for: Palliative care needs and preferences of female patients and their caregivers in Ethiopia: A rapid program evaluation in Addis Ababa and Sidama zone
Source: PLoS One. 2021 Apr 22;16(4):e0248738. doi: 10.1371/journal.pone.0248738 (PMC8062072; doi:10.1371/journal.pone.0248738)
Supplement: S1 Appendix — (DOCX) [file pone.0248738.s001.docx]

**Data extraction sheet – B4G and MJDA**

**Research ID:** ________________ **Patient ID:** ____________________ **Data entered in Excel by:** _______

**❑ Beza 4 generation**

**❑ Mary Joy**

Circle the code or fill out the blank box. 9 = missing data or unknown, yes = 1, no = 0.

1. **Sociodemographics**

| **Age (in years)** | **Nr of children** | **Nr of household members** | **Marital status** | **Religion** | **Subcity / Village** |
| --- | --- | --- | --- | --- | --- |
|  |  |  | \| 1. Single \| \| --- \| \| 2. Married \| \| 3. Separated \| \| 4. Widowed \| | \| 1. Orthodox \| \| --- \| \| 2. Christian, other \| \| 3. Muslim \| \| 4. Other \| |  |

1. **Economic and social assessment**

*(if known)*

| **Income** | **Housing** | **Occupation** | **Education** | **Care taker** | **Family support** |
| --- | --- | --- | --- | --- | --- |
|  |  |  |  |  |  |

1. **Initial presentation - management**

| **Opioids** | **Other analgesics** | **Steroids** | **Herbal medicine** |
| --- | --- | --- | --- |
| \| 0. no \| \| --- \| \| 1. yes \| \| 9. unknown \| | \| 0. no \| \| --- \| \| 1. yes \| \| 9. unknown \| | \| 0. no \| \| --- \| \| 1. yes \| \| 9. unknown \| | \| 0. no \| \| --- \| \| 1. yes \| \| 9. unknown \| |

1. **Referral and diagnosis**

| **Referred by** | **Reason for referral** | **Diagnosis** | **Metastasis** | **HIV-status** |
| --- | --- | --- | --- | --- |
| \| 1. CVW \| \| --- \| \| 2. Nurse \| \| 3. Self \| \| 4. Other: _________ \| | \| 1. pain relief \| \| --- \| \| 2. symptom relief \| \| 3. both pain and symptom relief \| \| 4. other: _________________ \| | \| 1. Cervix cancer \| \| --- \| \| 2. Breast cancer \| \| 3. Other: ________________ \| | \| 0. no \| \| --- \| \| 1. yes \| \| 9.unknown \| | \| 1. non-reactive \| \| --- \| \| 2. reactive \| \| 3. unknown \| |

1. **Pain on intake**

| **Pain present?** | **Relief by treatment at intake?** |
| --- | --- |
| Yes / no / unknown | 1 = None / 2 = partial / 3 = completely controlled |

1. **Treatment and support given**

| **Opioids** | **Other analgesics** | **Other medical treatment** | **Psychosocial support** | **Financial support** | **Spiritual support** |
| --- | --- | --- | --- | --- | --- |
| \| 0. no \| \| --- \| \| 1. yes \| \| 9. unknown \| | \| 0. no \| \| --- \| \| 1. yes \| \| 9. unknown \| | **1**  **2**  **3** | \| 0. no \| \| --- \| \| 1. yes \| \| 9. unknown \| | \| 0. no \| \| --- \| \| 1. yes \| \| 9. unknown \| | \| 0. no \| \| --- \| \| 1. yes \| \| 9. unknown \| |

1. **Pain on last month of care**

| **Pain present?** | **Relief by treatment?** |
| --- | --- |
| Yes / no / unknown | 1 = None / 2 = partial / 3 = completely controlled |

1. **Timeframe**

| Date of admission: | *(dd/mm/yy)* | Date of discharge: | *(dd/mm/yy)* |
| --- | --- | --- | --- |
| Date of death: | *(dd/mm/yy)* | Number of visits: | *(total)* |

**Data extraction sheet – Hospice Ethiopia**

**Research ID:** ________________ **Hospice ID:** ____________________ **Data entered in Excel by:** _______

Circle the code or fill out the blank box. 9 = missing data or unknown, yes = 1, no = 0.

1. **Sociodemographics** (page 1 and 4 of chart)

| **Age (in years)** | **Nr of children** | **Nr of household members** | **Marital status** | **Religion** | **Subcity** |
| --- | --- | --- | --- | --- | --- |
|  |  |  | \| 1. Single \| \| --- \| \| 2. Married \| \| 3. Separated \| \| 4. Widowed \| | \| 1. Orthodox \| \| --- \| \| 2. Christian, other \| \| 3. Muslim \| \| 4. Other \| | \| 1. Yeka \| \| --- \| \| 2. Gulele \| \| 3. Bole \| \| 4. Other \| |

1. **Economic and social assessment** (page 7 of chart)

Fill out the score (1 to 5)

| **Income** | **Housing** | **Occupation** | **Education** | **Family support** | **Total score** |
| --- | --- | --- | --- | --- | --- |
|  |  |  |  |  |  |

1. **Spiritual assessment** (page 4 of chart)

| **Is illness punishment of God?** | Yes / no / unknown |
| --- | --- |
| **Need support from spiritual fathers?** | Yes / no / unknown |

1. **Initial presentation – function assessment** (page 5 of chart)

Fill out the score (1 to 5)

| **Walking** | **Dressing** | **Toileting** | **Self care** | **Total score** |
| --- | --- | --- | --- | --- |
|  |  |  |  |  |

**Initial presentation - management** (page 2 and 3 of chart)

Fill out the score (1 to 5)

| **Opiods** | **Other analgesics** | **Steroids** | **Herbal medicine** |
| --- | --- | --- | --- |
| \| 0. no \| \| --- \| \| 1. yes \| \| 9. unknown \| | \| 0. no \| \| --- \| \| 1. yes \| \| 9. unknown \| | \| 0. no \| \| --- \| \| 1. yes \| \| 9. unknown \| | \| 0. no \| \| --- \| \| 1. yes \| \| 9. unknown \| |

1. **Referral and diagnosis** (page 1 and 2 of chart)

| **Referred by** | **Reason for referral** | **Diagnosis** | **Metastasis** | **HIV-status** |
| --- | --- | --- | --- | --- |
| \| 1. CVW \| \| --- \| \| 2. Nurse \| \| 3. Self \| \| 4. Other: _________ \| | \| 1. pain relief \| \| --- \| \| 2. symptom relief \| \| 3. both pain and symptom relief \| \| 4. other: _________________ \| | \| 1. Cervix cancer \| \| --- \| \| 2. Breast cancer \| \| 3. Other: ________________ \| | \| 0. no \| \| --- \| \| 1. yes \| \| 9.unknown \| | \| 1. non-reactive \| \| --- \| \| 2. reactive \| \| 3. unknown \| |

1. **Pain on intake** (page 6 of chart)

| **Pain nr** | **Pain present?** | **Score (0-5)** | **Relief?** |
| --- | --- | --- | --- |
| **1** | Yes / no / unknown |  | 1 = None / 2 = partial / 3 = completely controlled |
| **2** | Yes / no / unknown |  | 1 = None / 2 = partial / 3 = completely controlled |
| **3** | Yes / no / unknown |  | 1 = None / 2 = partial / 3 = completely controlled |
| **4** | Yes / no / unknown |  | 1 = None / 2 = partial / 3 = completely controlled |

1. **Treatment and support given** (page 11 and 12 of chart)

| **Opiods** | **Other analgesics** | **Other medical treatment** | **Spiritual support** |
| --- | --- | --- | --- |
| \| 0. no \| \| --- \| \| 1. yes \| \| 9. unknown \| | \| 0. no \| \| --- \| \| 1. yes \| \| 9. unknown \| | **1**  **2**  **3** | \| 0. no \| \| --- \| \| 1. yes \| \| 9. unknown \| |

1. **Pain on last month of care** (page 10? of chart)

| **Time** | **Pain present?** | **Score (0-5)** | **Relief?** |
| --- | --- | --- | --- |
| **Last month of care** | Yes / no / unknown |  | 1. = None / 2 = partial / 3 = completely controlled |

1. **Timeframe**

| Date of admission: | *(dd/mm/yy)* | Date of discharge: | *(dd/mm/yy)* |
| --- | --- | --- | --- |
| Date of death: | *(dd/mm/yy)* | Number of visits: | *(total)* |
