# Supplementary material for: Palliative care needs and preferences of female patients and their caregivers in Ethiopia: A rapid program evaluation in Addis Ababa and Sidama zone
Source: PLoS One. 2021 Apr 22;16(4):e0248738. doi: 10.1371/journal.pone.0248738 (PMC8062072; doi:10.1371/journal.pone.0248738)
Supplement: S2 Appendix — (DOCX) [file pone.0248738.s002.docx]

**Palliative Care Needs Assessment – patient survey**

*For researcher only:*

*Data entered on:*

*Initials:*

Date of interview:___________________ (dd/mm/yyyy) *[Gregorian calendar]*

Location of interview: ________________ Patient ID: _____________

**Patient information**

Age: _________years *[ estimate / checked in ID-card ]*

Village: ____________________

Palliative programme (circle): Hospice / B4G / MJDA

Location of nearest health center: ______________________________________

**1: General information – demographics and diagnosis**

| No | Question | Response |
| --- | --- | --- |
| 1 | How many people live in your house, including you? | ______________ (number) |
| 2 | How many of these are children (<15 years)? | ______________ (number) |
| 3 | What is your marital status?  *(circle one answer)* | 1. Single 2. Married 3. Separated or divorced 4. Widowed |
| 4 | What is the highest level of education you completed?  *(circle one answer)* | 1. Illiterate 2. Primary school 3. Secondary school 4. Higher than secondary school |
| 5 | What do you do for livelihood?  *(circle one answer)* | - 1. Unemployed   2. Work at own home / farmland   3. Daily labour, unskilled   4. Daily labour, skilled   5. Secure job   6. Unable to work because of illness   7. Unable to work because of high age |
| 5b. | Does it support your family and daily living expenses? | 1. Yes 2. Somewhat, but I need other source of income as well 3. No |
| 6 | What is your religion?  *(circle one answer)*  *Record particular church of Christianity, e.g. protestant, 7^th^ day Adventist* | 1. Orthodox 2. Other Christian,   specify ________________________   1. Muslim 2. Other: ________________________ |

**2: General information – diagnosis**

| No | Question | Response |
| --- | --- | --- |
| 7 | With what illness have you been diagnosed? |  |
| 8 | When were you diagnosed with this illness?  *Note if it is in Gregorian or Ethiopian calendar* | Month:  *Patient ID:*  Year: |
| 9 | Have you ever been tested for HIV?  *(circle one answer)*  *If ‘yes’ answer additional questions* | Yes   - Year of test: - Result: Positive / Negative / Unknown - If positive: using ARVs / not on ARVs   No |
| 10 | When did you join the palliative program?  *Note if it is in Gregorian or Ethiopian calendar* | Month:  Year: |
| 11 | What have you been told about your illness when you were diagnosed?  *Record all of the patients thoughts* |  |
| 12 | What did you feel when you were told about your diagnosis?  *Record all of the patients thoughts* |  |

**3: Signs and symptoms, including adjusted African Palliative Outcome Scale**

**
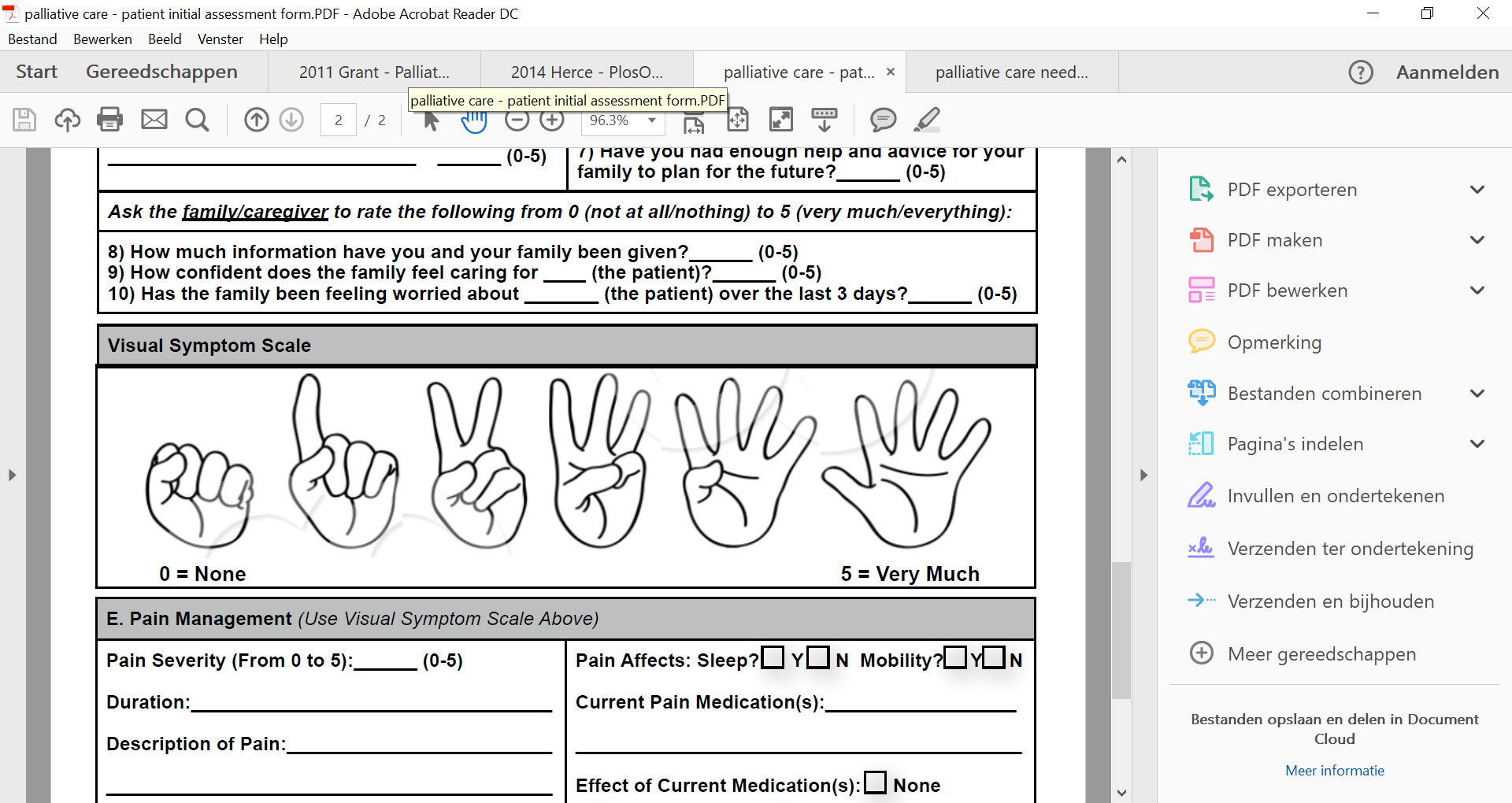
**

| No | Question | Response |
| --- | --- | --- |
| 13 | Could you rate your pain during the last one week?  *On a scale from 0 to 5*  *0 = no pain to 5 = overwhelming pain* | 0 - 1 - 2 - 3 - 4 - 5 |
| 14 | Have any other symptoms been affecting how you feel during the last one week?   - 1. Nausea or vomiting - 2. Constipation - 3. Diarrhoea - 4. Trouble eating - 5. Coughing - 6. Trouble breathing - 7. Trouble sleeping - 8. Other: _______________ - 9. Other: _______________ | *Patient ID:*  1. Yes / No If yes;__________ (0 – 5)  2. Yes / No If yes;__________ (0 – 5)  3. Yes / No If yes;__________ (0 – 5)  4. Yes / No If yes;__________ (0 – 5)  5. Yes / No If yes;__________ (0 – 5)  6. Yes / No If yes;__________ (0 – 5)  7. Yes / No If yes;__________ (0 – 5)  8. Yes / No If yes;__________ (0 – 5)  9. Yes / No If yes;-_________ (0 – 5) |
| 15 | Over the past week, have you been feeling worried about your health?  *On a scale from 0 to 5*  *0 = none to 5 = very much* | 0 - 1 - 2 - 3 - 4 - 5 |
| 16 | Over the past week, have you been able to share your concerns about your health with your family or friends? | Yes (1) / No (0) |
| 17 | Over the past week, how much did your illness affect your daily activities?  *On a scale from 0 to 5*  *0 = none to 5 = very much* | 0 - 1 - 2 - 3 - 4 - 5 |
| 18 | Over the past week, how much did your illness affect your social interaction?  *On a scale from 0 to 5*  *0 = none to 5 = very much* | 0 - 1 - 2 - 3 - 4 - 5 |

**4: Support and services**

| No | Question | Response |
| --- | --- | --- |
| 19a | What services do you need?  *(circle all answers that apply)*  *Allow first for spontaneous answers, before prompting options* | 1. Emotional support 2. Pain relief 3. Symptom relief (for e.g. nausea) 4. Spiritual support 5. Legal advice 6. Help making money / getting a job 7. Care for my children 8. Support with household tasks 9. Other: ____________________   __________________________ |
| 19b | Which of these services do you receive now? |  |
| 20a | What medical care and treatments are you receiving now?  *Ask patient*   - *what medication/treatments are for* - *who is providing care and treatment* | *Patient ID:* |
| 20b | What care and treatments did you receive before you joined the programme?  *Ask patient*   - *what medication/treatments are for* - *who is providing care and treatment* |  |
| 21 | Did you receive traditional or spiritual treatment, including ‘holy water’?  If yes, could you specify how it support you with your illness? | Yes (1) / No (0)  How does it support: |
| 22 | Do you receive support from an Iddir group?  If yes, could you specify how it support you with your illness? | Yes (1) / No (0)  How does it support: |
| 23 | Who is the person or people that help to take care of you at home?  *Record number of people and relation to patient* | ______________ (number)  Relation:   1. Sister / brother 2. Daughter / son 3. Other relative 4. Neighbour 5. Friend 6. Volunteer 7. Other _____________________ |
| 24 | From whom do you receive any kind of support? Can you describe the kind of support they give? (e.g. emotional, spiritual, financial)  *(circle all answers that apply and specify the kind of support)* | 1. Family inside the house   _____________________________  _____________________________   1. Family outside the house   _____________________________  _____________________________   1. Neighbours   _____________________________  _____________________________   1. Health Extension Worker   _____________________________  _____________________________   1. Palliative care program volunteer   _____________________________  _____________________________   1. Iddir group   *Patient ID:*  _____________________________  _____________________________   1. Church or religious group   _____________________________  _____________________________   1. Others:   _____________________________  _____________________________  _____________________________  _____________________________  _____________________________ |
| 25 | Can you name the 3 most important support groups that support you in your community? | 1.  2.  3. |
| 26 | What services do you need but you are not receiving now?  *(circle all answers that apply)*  *Allow first for spontaneous answers, before prompting options* | 1. Emotional support 2. Pain relief 3. Symptom relief (for e.g. nausea) 4. Spiritual support 5. Legal advice 6. Help making money / getting a job 7. Care for my children 8. Support with household tasks 9. Other: ____________________   __________________________ |
| 27 | What are barriers for you to access these services?  *Open question (prompt with e.g. distance to facility, financial constraints)* |  |
| 28 | Can you describe what palliative care is in your own words? |  |
| 29 | Did you plan about your end of life?  *(If no, continue directly to question 32)* | Yes (1) / No (0) |
| 30 | What did you plan about your end of life? | *Patient ID:* |
| 31 | Who supports you in the planning?  *(after this question continue to question 33)* |  |
| 32 | *If no in question 29*, why did you not plan about it? |  |
| 33 | If you had the opportunity to choose, where would you most like to receive palliative care?  *Explain all options carefully.*  *Ask about the reasons why this option has their preference.* | 1. At home 2. At the nearby health center 3. At the hospital 4. At NGO facility 5. Other: _______________________ 6. I have no preference   Reason: ________________________ |
| 34 | Do you have any other comments? |  |
| 35 | Do you have any questions? |  |

***Thank you for your time and participation!***

**For interviewer:**

Could the interview be completed without interruption?

yes / no if ‘no’ specify ___________________________________________________

Did translation affect the quality of the interview?

yes / no if ‘yes’ specify ___________________________________________________

Did a hearing problem affect the quality of the interview?

yes / no if ‘yes’ specify ___________________________________________________

Other comments:

Initials interviewer: __________________ Initials translator: _______________
