# Supplementary material for: Palliative care needs and preferences of female patients and their caregivers in Ethiopia: A rapid program evaluation in Addis Ababa and Sidama zone
Source: PLoS One. 2021 Apr 22;16(4):e0248738. doi: 10.1371/journal.pone.0248738 (PMC8062072; doi:10.1371/journal.pone.0248738)
Supplement: S3 Appendix — (DOCX) [file pone.0248738.s003.docx]

**Palliative Care Needs Assessment – caregiver survey**

*For researcher only:*

*Data entered on:*

*Initials:*

Date of interview:___________________ (dd/mm/yyyy) *[Gregorian calendar]*

Location of interview: ________________ Caregiver ID: _____________

**Caregiver information**

Gender (circle): Male / Female

Age: _________years *[ estimate / checked in ID-card ]*

Village: ____________________

Palliative programme (circle): Hospice / B4G / MJDA

Location of nearest health center: ______________________________________

**1: General information – demographics and diagnosis**

| No | Question | Response |
| --- | --- | --- |
| 1 | How many people live in your house, including you? | ______________ (number) |
| 2 | How many of these are children (<15 years)? | ______________ (number) |
| 3 | What is your marital status?  *(circle one answer)* | 1. Single 2. Married 3. Separated or divorced 4. Widowed |
| 4 | What is the highest level of education you completed?  *(circle one answer)* | 1. Illiterate 2. Primary school 3. Secondary school 4. Higher than secondary school |
| 5 | What do you do for livelihood?  *(circle one answer)* | - 1. Unemployed   2. Work at own home / farmland   3. Daily labour, unskilled   4. Daily labour, skilled   5. Secure job   6. Unable to work because of illness   7. Unable to work because of high age |
| 5b. | Does it support your family and daily living expenses? | 1. Yes 2. Somewhat, but I need other source of income as well 3. No |
| 6 | What is your religion?  *(circle one answer)*  *Record particular church of Christianity, e.g. protestant, 7^th^ day Adventist* | 1. Orthodox 2. Other Christian,   specify ________________________   1. Muslim 2. Other: ________________________ |
| 7 | What is your relationship to the patient you care for? |  |
| 8 | What is the primary diagnosis of the patient you care for? |  |

**2: adjusted African Palliative Outcome Scale**

**
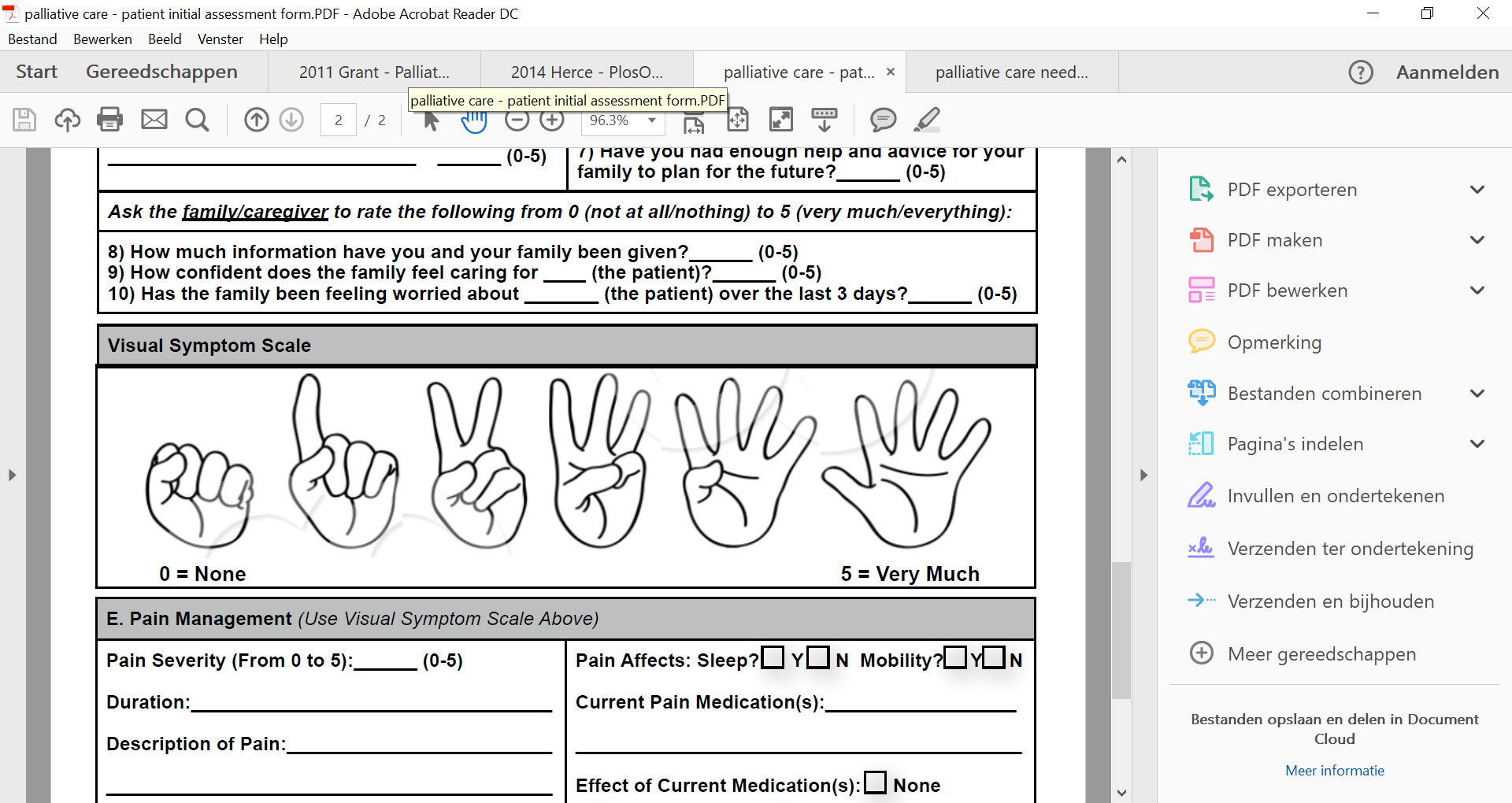
**

| No | Question | Response |
| --- | --- | --- |
| 9 | Over the past one week, how confident have you felt caring for the patient?  *On a scale from 0 to 5*  *0 = none to 5 = very much* | 0 - 1 - 2 - 3 - 4 - 5 |
| 10 | Over the past one week, have you been feeling worried about the patient?  *On a scale from 0 to 5*  *0 = none to 5 = very much* | 0 - 1 - 2 - 3 - 4 - 5 |

**3: Care giving, support and needs**

| No | Question | Response | |  |
| --- | --- | --- | --- | --- |
| 11 | How long have you been providing care for this patient? | _______ days  _______ months  _______ years | |  |
| 12 | How did you come to care for this patient?  *(circle all answers that apply)* | 1. We are relatives 2. The patient is my friend 3. The patient lives in my neighbourhood 4. I volunteered 5. I am the only one available to care for her 6. The patient was abandoned 7. Other: _________________________ | |  |
| 13 | How many hours a day do you provide care to this patient?  *Quantify the number of waking hours that the person spends caring for the patient.*  *Put 24 hours if a patient is completely unable to care for themselves.* | _______ hours | |  |
| 14 | What exactly do you do in caring for this patient?  *(circle all answers that apply)* | 1. Feed the patient 2. Bath the patient 3. Give medication 4. Dress the patient 5. Transport patient to the hospital or clinic 6. Provide care for their children 7. Provide emotional support 8. Provide housing 9. Provide monetary support 10. Other: ______________________ | |  |
| 15 | Do you receive support from anyone in caring for this patient? | Yes (1) / No (0) | |  |
| 16 | If yes, by whom?  *(note the relationship to the caregiver or patient, e.g. friend of caregiver, sister of patient)* |  | |  |
| 17 | What kind of support do you receive from the person named in question 16? |  | |  |
| 18 | Have you received any training to support you in caring for the patient? | | Yes (1) / No (0) | |
| 19 | If yes, by whom?  *(note the name of organization or function of the person)* | |  | |
| 20 | Did the training provide you with the skills you need? | | 1. Yes 2. Somewhat 3. No 4. Don’t know | |
| 21 | Have you taken care of other severely sick patients?  *If ‘no’ continue to question 23* | | Yes (1) / No (0) | |
| 22 | What type of care did you provide before? | | 1. Feed the patient 2. Bath the patient 3. Give medication 4. Dress the patient 5. Transport patient to the hospital or clinic 6. Provide care for their children 7. Provide emotional support 8. Provide housing 9. Provide monetary support 10. Other: ______________________ | |
| 23 | What major problems do you encounter when you look after your current patient?  *(circle all answers that apply)* | | 1. It is very hard work 2. I do not have any help 3. I do not know how to provide care 4. I do not have enough money 5. I do not have time for other work to make money or provide food for my family 6. It makes me sad 7. I worry about the future 8. I feel stigmatized by the community 9. Other: ________________________ | |
| 24 | What are your main reasons to continue caring for this patient?  *(circle all answers that apply)* | | 1. We are relatives 2. The patient is my friend 3. I am the only one available to care for her 4. It is rewarding to me 5. My family expects me to care for her 6. I cannot get a job 7. Other: _________________________ | |
| 25 | Have you received psychological of spiritual support when you need it? | | 1. Yes 2. No 3. I did not need this support | |
| 26 | If yes, by whom?  *(note the name of organization or relation to the person)* | |  | |
| 27 | Do you receive support from an Iddir group?  If yes, could you specify how it supports you? | | Yes (1) / No (0)  How does it support: | |
| 28 | How do you perceive your own health?  *(Circle one answer)* | | 1. Very poor 2. Poor 3. Neither poor nor good 4. Good 5. Very good | |
| 29 | What have you been told by the patient or his/her relatives about his/her illness?  *Record all of the caregivers thoughts* | |  | |
| 30 | How do you feel about his/her illness?  *Record all of the caregivers thoughts* | |  | |
| 31 | What are your greatest fears now?  *Circle all that apply, carefully read all options* | | 1. The patient being in pain 2. Losing the patient I am caring for 3. Not having enough money to provide care 4. Not knowing how best to provide care 5. Not being able to provide good care in the home situation 6. Not receiving help from others 7. Being criticized by others 8. My family’s health 9. Not being able to work for income 10. Not having plans for my own future 11. Other: _________________________ | |
| 32 | What assistance would make it easier for you to look after this patient?  *Circle all that apply, carefully read all options* | | 1. Support with food 2. Monetary support 3. Income generating activity 4. Caregiving supplies (like gloves) 5. Affordable medical care 6. Support from others in caretaking 7. Training in patient care 8. Peer group with other caregivers 9. Other: _______________________ | |
| 32b | What are barriers for you and your patient to access these palliative care services?  *Open question (prompt with e.g. distance to facility, financial constraints)* | |  | |
| 33 | Can you describe what palliative care is in your own words? | |  | |
| 34 | Do you plan with the patient you are caring for about end of life? | | Yes (1) / No (0) | |
| 35 | *If yes to question 34,* what have you planned about end of life? | |  | |
| 36 | *If no to question 34*, why have you not planned about end of life? | |  | |
| 37 | If you had the opportunity to choose, where would you prefer this patient to receive care?  *Explain all options carefully.*  *Ask about the reasons why this option has their preference.* | | 1. At home 2. At the nearby health center 3. At the hospital 4. At NGO facility 5. Other: _______________________ 6. I have no preference   Reason: ________________________ | |
| 38 | Do you have any other comments? | |  | |
| 39 | Do you have any questions? | |  | |

***Thank you for your time and participation!***

**For interviewer:**

Could the interview be completed without interruption?

yes / no if ‘no’ specify ___________________________________________________

Did translation affect the quality of the interview?

yes / no if ‘yes’ specify ___________________________________________________

Did a hearing problem affect the quality of the interview?

yes / no if ‘yes’ specify ___________________________________________________

Other comments:

Initials interviewer: __________________ Initials translator: _______________
