# Supplementary material for: Palliative care needs and preferences of female patients and their caregivers in Ethiopia: A rapid program evaluation in Addis Ababa and Sidama zone
Source: PLoS One. 2021 Apr 22;16(4):e0248738. doi: 10.1371/journal.pone.0248738 (PMC8062072; doi:10.1371/journal.pone.0248738)
Supplement: S4 Appendix — (DOCX) [file pone.0248738.s004.docx]

**Palliative Care Needs Assessment – stakeholder survey**

*For researcher only:*

*Data entered on:*

*Initials:*

Date of interview:___________________ (dd/mm/yyyy) *[Gregorian calendar]*

Location of interview: ________________

**Stakeholder information**

Name: ________________________________________

Gender (circle): Male / Female

Age: _________years *[ estimate / checked in ID-card ]*

Function: _______________________________________

Organization: ____________________________________

**1: General introduction**

| No | Question | Response |
| --- | --- | --- |
| 1 | Can you describe what palliative care is, in your own words? |  |
| 2 | What are in your opinion the components of palliative care? |  |
| 3 | How are you involved in palliative care? |  |

*Stakeholder ID:*

**2: Program and activities**

***For stakeholders involved in a palliative care or support care program, continue below with question 4 – 14***

*For stakeholders involved in a community network / religious organization continue to question 15****.***

| No | Question | Response |
| --- | --- | --- |
| 4 | How is your organization involved in palliative care? |  |
| 5 | What services are provided for patients under the program? |  |
| 6 | Who is providing these services? |  |
| 7 | What training did the providers receive?  *(note by whom training was provided)* |  |
| 8 | Based on what criteria are patients included in the program? |  |
| 9 | Who can refer to the program? |  |
| 10 | Do you work together with other health care providers?  *If yes, who?* | *Stakeholder ID:* |
| 11 | Do you work together with other organizations or programs?  *If yes, who?* |  |
| 12 | What are the strong points of the program? |  |
| 13 | What would you like to improve about the program? |  |
| 14 | Are you aware of the national protocol on palliative care?  *If yes, which version?* |  |

***For stakeholders involved in a community network / religious organization: question 15 - 26***

| No | Question | Response |
| --- | --- | --- |
| 15 | How is your organization involved in palliative care? |  |
| 16 | What services are provided in your community to severely ill patients? | *Stakeholder ID:* |
| 17 | Who is providing these services? |  |
| 18 | What training did the providers receive?  *(note by whom training was provided)* |  |
| 19 | Who can access these services? |  |
| 20 | How are family members of severely ill patients supported? |  |
| 21 | How does the community view severely ill patients? |  |
| 22 | How does the community approach family of severely ill patients? |  |
| 23 | Do you work together with health care providers?  *If yes, who?* |  |
| 24 | Do you work together with other organizations or programs?  *If yes, who?* | *Stakeholder ID:* |
| 25 | What are the strong points of the program? |  |
| 26 | What would you like to improve about the program? |  |

**3: Barriers and priorities in palliative care**

***For all stakeholders***

| No | Question | Response |
| --- | --- | --- |
| 27 | What are barriers for patients to access palliative care? |  |
| 28 | What are current gaps you experience in palliative care? |  |
| 29 | What are priorities for improving palliative care? |  |
| 30 | Who should be responsible to work on these priorities? | *Stakeholder ID:* |
| 31 | What are your expectations for palliative care in the future? |  |
| 32 | Do you have any other comments? |  |
| 33 | Do you have any questions? |  |

***Thank you for your time and participation!***

**For interviewer:**

Could the interview be completed without interruption?

yes / no if ‘no’ specify ___________________________________________________

Did translation affect the quality of the interview?

yes / no if ‘yes’ specify ___________________________________________________

Did a hearing problem affect the quality of the interview?

yes / no if ‘yes’ specify ___________________________________________________

Other comments:

Initials interviewer: __________________ Initials translator: _______________
