# Supplementary material for: Palliative care needs and preferences of female patients and their caregivers in Ethiopia: A rapid program evaluation in Addis Ababa and Sidama zone
Source: PLoS One. 2021 Apr 22;16(4):e0248738. doi: 10.1371/journal.pone.0248738 (PMC8062072; doi:10.1371/journal.pone.0248738)
Supplement: Attachment — Submitted filename: PONE-D-20-13293R1 Point by point response.docx [file pone.0248738.s006.docx]

**Point by point response**

**PONE-D-20-13293R1**

**Palliative care needs and preferences of female patients and their caregivers in Ethiopia:**

**a rapid program evaluation in Addis Ababa and Sidama zone**

| **Technical comment** | **Authors’ response** |
| --- | --- |
| 1. **ORCiD iD**   It appears that your ORCiD iD has not been validated in your Editorial Manager account and we are unable to proceed until that step is complete. | Thank you very much. Comment accepted and  We have authenticated the iD. |
| 2. **Ethics statement**   Your ethics statement must appear in the Methods section of your manuscript. If your ethics statement is written in any section besides the Methods, please move it to the Methods section and delete it from any other section. Please also ensure that your ethics statement is included in your manuscript, as the ethics section of your online submission will not be published alongside your manuscript. | Thank you. We have accepted and move the ethics statement from the declaration part to method section in the revised manuscript (Method section, from line 189 to line 200, page 08). We also ensure that the ethics statement included in the manuscript is similar with the ethics section in the online submission. |
