# Supplementary material for: Palliative care needs and preferences of female patients and their caregivers in Ethiopia: A rapid program evaluation in Addis Ababa and Sidama zone
Source: PLoS One. 2021 Apr 22;16(4):e0248738. doi: 10.1371/journal.pone.0248738 (PMC8062072; doi:10.1371/journal.pone.0248738)
Supplement: Attachment — Submitted filename: PONE-D-20-13293R1 Response to reviewers.docx [file pone.0248738.s007.docx]

**Response to reviewers PONE-D-20-13293R1**

Have the authors made all data underlying the findings in their manuscript fully available?
The Plos data policy requires authors to make all data underlying the findings described in their manuscript fully available without restriction, with rare exception (please refer to the Data Availability Statement in the manuscript PDF file). The data should be provided as part of the manuscript or its supporting information, or deposited to a public repository. For example, in addition to summary statistics, the data points behind means, medians and variance measures should be available. If there are restrictions on publicly sharing data e.g. participant privacy or use of data from a third party—those must be specified.

**Reviewer # 2 comments: No**

**Reply to Reviewer 2 comments**

- *Thank you. The data is fully accessible as supporting information and also it can be available from the corresponding author upon reasonable request.*

**Reviewer #2:** Thank you addressing previous comments in this current revision. Most of my queries have been addressed, but it would be helpful to include aspects of your response into the body of the manuscript. For example, thank you for clarification on the way in which you sought to capture a representative sample. However, it would be helpful for readers of the manuscript if this rationale outlined in your response was outlined in the body of the manuscript so that your decision making around accessing the 10 – 15% of the total case load is clear.

**Reply to Reviewer 2 comments**

- *Thank you for the additional suggestion to provide the rationale outlined in the response in the body of the manuscript to clarify on accessing 10-15% of the total case load. We provided the rationale in the method section (Method section, page 5&6, from line 120 to 127)*

**Reviewer #2:** Similarly, your mention of continuously monitoring the data during the project (to determine whether new themes were emerging) would be useful to add further transparency to the approach adopted.

**Reply to Reviewer 2 comments**

- *Thank you for this useful insight. Here we were referring to the research process as project, which we realized may give a wrong impression. The point here has to do with the fact that we developed themes based on the research questions before the data collection. During the data collection as well as transcription, we kept note of emerging themes and/or if pre-defined themes still are valid based on evidences generated from the data. The analysis thus follows the themes that were finalized following completion of data collection (Method section, page 7, from line 183 to 186).*

**Reviewer #2:** Thank you for clarifying that you adopted an inductive approach to framework analysis. Please can you add the term ‘inductive’ into the body of the manuscript. I was, however, unclear on how pre-identified codes were used within the inductive approach. This may be an issue with terminology, but please can you provide more detail about the use of pre-identified codes.

**Reply to Reviewer 2 comments**

- *Thank you again for this useful point. We fully agree this is a bit confusing. Codes were developed early on before data collection based on the research question. These codes were finalized later based on data generated and transcribed. The coding of interview data was made by two researchers [MK and KS] with support from [MF] in cases of discrepancies. While the pre-coding is possible that doesn’t warrant inductive approach. So, this is removed and corrections made accordingly (Method section, page 7&8, from line 181 to 187)*

**Reviewer #2:** Please add details into the manuscript regarding which caregivers were involved in the interviews, and details around interviews with patient and caregiver participants being conducted alone, as you have outlined in your response.

**Reply to Reviewer 2 comments**

- *Thank you for this insight which will make the reading clearer. We included this information in the revised manuscript (Method section, page 6, from line 128 to 140. Additional details about how interviews with patient and caregiver participants being conducted has also provided in data collection and ethics sections.*

**Reviewer #2:** The recommendations are very specific, aligning with a proposed research design or an awareness strategy. Is it possible to broaden the recommendations to consider, for example, how the findings align with the pillars of palliative care (i.e. policy, education, drug availability, implementation, and research) or policy relating to provision of care in Ethiopia?

**Reply to Reviewer 2 comments**

- *We appreciate for this. We considered broadening the recommendation has an added value to the research outcome that we broadened the recommendations based on the study finding (Recommendation section, page 22, from line 484 to 498)*
