# Supplementary material for: Palliative care needs and preferences of female patients and their caregivers in Ethiopia: A rapid program evaluation in Addis Ababa and Sidama zone
Source: PLoS One. 2021 Apr 22;16(4):e0248738. doi: 10.1371/journal.pone.0248738 (PMC8062072; doi:10.1371/journal.pone.0248738)
Supplement: Attachment — Submitted filename: PONE-D-20-13293R2 Response to reviewers.docx [file pone.0248738.s008.docx]

**Response to reviewers PONE-D-20-13293R2**

Dear Editor,

We would like to thank you for providing us with useful comments and queries. We found this a useful opportunity to improve the quality of our submission to the expected standard of your journal. More particularly, the comments and queries helped us to get back to our data and check details of the methods we used. Below, you will find point-by-point clarifications and explanations.

Thank you again,

Kalkidan Solomon

On behalf of all authors.

**Editor Comments:**
Like Reviewer #2, I would like to question the part of your Methods where contrast your 'mixed methods' approach with a 'primarily qualitative approach'. Please provide more information about the type of mixed methods you employed according to a recognized typology, such as Creswell's (2018), including details of how quantitative and qualitative data were integrated. As they are currently written, your Methods and Results look very much like a qualitative study, where the quantitative data were used only to describe the sample and nothing more?

**Reply to editor comments**

- *Thank you for this useful point. We fully agree this study is largely qualitative. Nonetheless, there are specific research questions that necessitated both qualitative and quantitative approach. we employed concurrent parallel design (Creswell and Creswell 2017) Both qualitative and quantitative data were collected in parallel and are both part of the rapid program evaluation approach as described in the method section (method section, page 5 from line 98 to 100). The quantitative data were used to describe such findings as palliative outcome scale score (POS) to express the severity of symptoms among patients, to quantify preference for institutional or home based care, to describe how many of the participants had end of life plan etc (method section, page 6, from line 152 to 155).*

**Editor Comments:**

On a related point, while you indicate that you did not use saturation to inform sample size, you don't suggest what alternative method was used beyond specifying what appears to be an arbitrary 10-15% 124 of the total case load of each program?

**Reply to editor comments**

*Thank you for your request to further specify our sample size selection. We want to clarify here that saturation was not strictly defined on how to track at the beginning. However, with 8-12 participants from the respective sites, saturation was achieved after the fifth participant was interviewed. We interviewed additional participants after presumed saturation was achieved to ascertain repetition of evidence were beyond doubt. In this research evidence was triangulated by different sources and methods, that makes the data presented in this manuscript very strong (method section, page 5 & 6, from line 121 to 136).*

**Reviewer # 2 comments:**

I am keen to ensure transparency in the reporting of the methods around your handling of the interview data. I appreciate that this was a rapid program evaluation, but the methods still need to be very clear. Previous revisions have helped to clarify the approach you have taken, but following your last response (removal of the 'inductive' element) I am now unsure of which approach was adopted. Please can you clarify, was this a deductive thematic analysis that you adopted? And if so, please can you provide a supporting citation of the analysis approach, such as Braun and Clarke.

**Reply to Reviewer 2 comments**

*Thank you as well for this useful point. As pointed out above, it is true that this study is largely qualitative. Nonetheless, there are specific research question that necessitated quantitative evidence. The findings however remain descriptive that we were guided by inductive approach. Based on your previous and current comments we explained this in the body of the manuscript under method section, page 7, line 182. Qualitative data coding was developed early on before data collection based on the research question. However, these codes were refined later following transcription of data. Eventual coding of the data was made by two researchers [MK and KS] with support from [MF] in cases of discrepancies. Quantitative data helped to describe palliative outcome scale score (POS) to express the severity of symptoms among patients, to quantify preference for institutional or home based care, to describe how many of the participants had end of life plan etc that could not be addressed through qualitative method (method section, page 6, from line 152 to 155, result section, page 9 from line 233 to 241, page 12 from line 297 to 319). As it is presented in the result section qualitative data helped to explain physical signs and symptoms, perceived challenges and preferences for palliative care, end-of-life planning. perceived support, preferences for provision of palliative care, existing health service activities, gaps and challenges in palliative care programs.*

**Reviewer #2** **comments:**

In your most recent response, you have outlined that the objective of your approach to interviewing was unlike other qualitative research where the focus is on achieving data saturation I would strongly disagree with this being the only means of determining the completeness and quality of data captured in a qualitative study and would suggest that this statement is removed or revised

**Reply to Reviewer 2 comments**

*We agree with your insightful challenge and we revised the statement (Method section, page 5, from line 121 to 127).*

**Reference**

Creswell, J. W. and J. D. Creswell (2017). Research design: Qualitative, quantitative, and mixed methods approaches, Sage publications.
