# Supplementary material for: Palliative care needs and preferences of female patients and their caregivers in Ethiopia: A rapid program evaluation in Addis Ababa and Sidama zone
Source: PLoS One. 2021 Apr 22;16(4):e0248738. doi: 10.1371/journal.pone.0248738 (PMC8062072; doi:10.1371/journal.pone.0248738)
Supplement: Attachment — Submitted filename: Response to reviewer PONE-D-20-13293R4.docx [file pone.0248738.s009.docx]

**Response to Editor PONE-D-20-13293R4**

Dear Editor,

### We would like to thank you for providing us with useful comments. We revised the data availability statement in the manuscript.

Thank you

Kalkidan Solomon

On behalf of all authors.
