# Supplementary material for: Palliative care needs and preferences of female patients and their caregivers in Ethiopia: A rapid program evaluation in Addis Ababa and Sidama zone
Source: PLoS One. 2021 Apr 22;16(4):e0248738. doi: 10.1371/journal.pone.0248738 (PMC8062072; doi:10.1371/journal.pone.0248738)
Supplement: Attachment — Submitted filename: Response to reviewers-PONE-D-20-13293-R4.docx [file pone.0248738.s010.docx]

**Response to Editor PONE-D-20-13293R4**

# On behalf of all authors We thank you for your constructive feedback on our manuscript entitled **“Palliative care needs and preferences of female patients and their caregivers in Ethiopia; a rapid program evaluation” [PONE-D-20-13293R4].** Please find the point by point responses here below.

We trust that the improvements suggested by both academic editors and reviewers made this revised manuscript suitable for publication in PLOS ONE. We are looking forward to hearing from you.

Yours sincerely,

Kalkidan Solomon

On behalf of all authors

**Editor Comments**

1. Thank you for your response, which clarifies the third question asked on my last review. However, I'm afraid you still haven't responded satisfactorily to the first and second questions. No changes have been made to clarify the method used for integration or - if no such methods were used - to change the approach from mixed methods to qualitative (noting that some survey information was also collected from participants to describe the sample).

- Thank you for this useful point. Our study applied rapid evaluation methodology with mixed method approach. This study is largely qualitative. On the other hand, there are specific research questions that necessitated description using quantitative approach. Both qualitative and quantitative data were collected in parallel and are both part of the rapid program evaluation approach. As it is presented in the result section qualitative data alone helped to explain vast majority of the study objectives including; physical signs and symptoms, perceived challenges and preferences for palliative care, end-of-life planning. perceived support, preferences for provision of palliative care, existing health service activities, gaps and challenges in palliative care programs. But the findings of quantitative study however remain descriptive and we didn’t have integration of both qualitative and quantitative finding for a single objective since we have used quantitative data mainly to describe/quantify palliative outcome scale score among (POS) to express the severity of symptoms among patients.

2. Also, your confirmation that codes were predefined prior to analysis means the approach can no longer be described as 'inductive' throughout. Might an integrated approach of the kind described by Bradley et al (2007) be more fitting, for example <https://onlinelibrary.wiley.com/doi/pdf/10.1111/j.1475-6773.2006.00684.x> ?

- Thank you for your suggestion. We analyzed the interview data inductively. As we mentioned earlier we had predefined code/ codebook prior to analysis based on the objectives but we have made a lot of amendments in to the code, we added new coded basing what we got from the data so we were free to have emerging codes from the data and all the codes, categories and themes in this study were emerging from the data itself and that is why we reported as we analyzed the interview data inductively. To avoid ambiguity in this regard and since we practically follow inductive approach we have removed the statement which stated that “predefined codes were used during analysis from the manuscript and replaced with the statement as ‘we analyzed the data inductively’ (Method section, Page 7, from line 183 to 184)
