# Supplementary material for: Palliative care needs and preferences of female patients and their caregivers in Ethiopia: A rapid program evaluation in Addis Ababa and Sidama zone
Source: PLoS One. 2021 Apr 22;16(4):e0248738. doi: 10.1371/journal.pone.0248738 (PMC8062072; doi:10.1371/journal.pone.0248738)
Supplement: Attachment — Submitted filename: Response to reviewers-PONE-D-20-13293-R5.docx [file pone.0248738.s011.docx]

**Response to Editor PONE-D-20-13293R5**

# On behalf of all authors We thank you for your constructive feedback and suggestions on our manuscript entitled **“Palliative care needs and preferences of female patients and their caregivers in Ethiopia; a rapid program evaluation” [PONE-D-20-13293R5].** Please find the point by point responses here below.

We trust that the improvements suggested by both academic editors and reviewers made this revised manuscript suitable for publication in PLOS ONE. We are looking forward to hearing from you.

Yours sincerely,

Kalkidan Solomon

On behalf of all authors

**Editor Comment**

1. However, I am now even less satisfied that the overall approach taken can be described as mixed methods given that the authors have confirmed that no integration occurred. According to Creswell and all other leading methodologists I am aware of, integration is a requirement of mixed methods. Please either provide evidence from the literature that the approach taken meets the requirements of mixed methods without integration or else change the nomenclature throughout to describe the study as using a qualitative approach, while also collecting some quantitative data.

- Thank you for your suggestion. As we explained before this study is largely qualitative. By now we understand and accept your suggestion and changed the terminology throughout the manuscript to describe the study as using a qualitative approach, while also (as it is presented in the result section) the descriptive findings of quantitative study were used to describe/quantify palliative outcome scale score (POS) to express the severity of symptoms among patients. Thus we removed the content from the manuscript that might be confusing to readers in this regard. (Abstract section, Page 2 and Method Section Page 5 & 6).
