# Supplementary material for: Palliative care needs and preferences of female patients and their caregivers in Ethiopia: A rapid program evaluation in Addis Ababa and Sidama zone
Source: PLoS One. 2021 Apr 22;16(4):e0248738. doi: 10.1371/journal.pone.0248738 (PMC8062072; doi:10.1371/journal.pone.0248738)
Supplement: Attachment — Submitted filename: Response to reviewers-PONE-D-20-13293-R6.docx [file pone.0248738.s012.docx]

**Response to Editor PONE-D-20-13293R6**

# On behalf of all authors We thank you for your constructive feedback and suggestions on our manuscript entitled **“Palliative care needs and preferences of female patients and their caregivers in Ethiopia; a rapid program evaluation” [PONE-D-20-13293R6].** Please find the point by point responses here below.

We trust that the improvements suggested by both academic editors and reviewers made this revised manuscript suitable for publication in PLOS ONE. We are looking forward to hearing from you.

Yours sincerely,

Kalkidan Solomon

On behalf of all authors

**Editor Comment**

**Abstract**
1. The abstract in the online system still says ‘mixed methods’ even though this has been changes to qualitative in the manuscript; please harmonise.

- Thank you. We harmonized the online content with the manuscript content

2. The methods should state that the POS was administered.

- Thank you for your suggestion. We included that POS was used to describe physical symptoms in the abstract (Abstract section, from line 38 to 40).

3. Please reword the sentence on analysis to state: ‘Descriptive analyses were used for POS data, and an inductive thematic analysis for the interview data’.

- Thank you again for your suggestion. We reword and add statement which describe about POS data and inductive thematic analysis for the qualitative data (Abstract section, from line 38 to 40).

4. The sentence in the conclusion that begins ‘pain was poorly controlled’ really just repeats what was in the results and should be removed

- We accept the comment and revised accordingly. Thank you (Abstract section, from line 50 to 52)

**Manuscript**
1. Please ensure ‘a’ is inserted before ‘qualitative study/approach’ throughout.

- Thank you for your suggestion. We just added ‘a’ before ‘qualitative study/approach’ throughout.

2.Please remove the following sentence which unnecessarily repeats: ‘Without integration with qualitative data, the quantitative data alone were used to describe palliative outcome scale score (POS) to express the severity of symptoms among patients’.

- Thank you for your feedback. We removed the statement (Method section, from line 150 to 152)

3. Please ensure the abbreviation POS is used throughout without the full name after this has been introduced the first time.

- Thank you. We revised and used the abbreviation ‘POS’ throughout without the full name after POS has been introduced the first time.

4. Cut-offs for the POS should be moved from the Results to the Methods and justified with a reference. Rather than use ‘low’ and ‘high’ which are ambiguous, I suggest just sticking with ‘none-mild’ and ‘moderate-severe’.

- Thank you for the valuable comments. We have moved Cut-offs for the POS to method section and added reference. In addition, ‘low and high’ words were removed to avoid confusion and as suggest we used ‘none-mild’ and ‘moderate-severe’ (Method section, from line 175 to 180 and Result section, from line 234 to 236 and Table 3, from line 626 to 629).

5. Please list the themes in sentence format rather than list form, i.e. ‘We identified the following themes in the semi-structured interview transcripts: awareness of palliative care; organization of palliative care and referral pathways …’

- We accept the suggestion and revised accordingly. Thank you (Result section, from line 244 to 256)

6. For Table 1, give the overall number of participants at the end of the title in brackets (i.e ‘(N=XX’).

- Thank you for your suggestion. We add the total number of participants for both table one two (Table section, line 630 and line 637)

7. Don’t repeat the contents of Table 1 in the text but only include any information that is additional.

- We accept the feedback and revised the text accordingly. Thank you (Result section, from line 220 to 228).

8. Remove ‘age in years, median’ from Table 1 as it’s in the text and disrupts the column headings.

- Thank you. As suggested, we removed the row from Table 1 (Table 1, line 630).

9. Move the contents of Table 2 to Table 1, as these are continuing socio-demographic characteristics.

- Thank you for your suggestion. We presented the socio-demographic characteristics of both patients and caregivers in one table when we initially submitted this manuscript but both reviewers (#1 and 2) commented on it to split the table in to two and present the socio-demographic characteristics of the patients and caregivers in separate table then based on reviewers comment we split and presenting using two table.

10. Include an explanation in the text of why so much data were missing

- Thank you for your suggestion. In this study a total of 27 care givers were included (12 with primary caregivers, 15 with voluntary caregivers) and as total number was indicated in the table we took the socio-demographic profile from all caregivers who were included in this study but we only select variables which had links with the study objectives. Nonetheless. nothing was missed while we collected the data.
